# Designing a Pest and Disease Outbreak Warning System for Farmers, Agronomists and Agricultural Input Distributors in East Africa

**DOI:** 10.3390/insects13030232

**Published:** 2022-02-26

**Authors:** Molly E. Brown, Stephen Mugo, Sebastian Petersen, Dominik Klauser

**Affiliations:** 1 6th Grain Corporation, Bethesda, MD 20817, USA; 2 Center for Resilient Agriculture for Africa (CRA-Africa), Nairobi, P.O. Box 286, Kiserian 00206, Kenya; mugosn58@gmail.com; 3 Syngenta Foundation for Sustainable Agriculture, 4058 Basel, Switzerland; syngenta.foundation@syngenta.com (S.P.); dominik.klauser@syngenta.com (D.K.)

**Keywords:** fall armyworm, early warning system, maize, Kenya, Africa, cultural control

## Abstract

**Simple Summary:**

Designing early warning systems for threats to food crops in Africa must respond to the needs of potential users of the system. This paper provides evidence from professional distributors, retailers, researchers, and agronomists in East Africa who may be able to use and communicate the results of the predictive modeling of pest outbreaks. Understanding the timing and spatial extent of required warnings will help guide research and engagement in these rapidly commercializing countries.

**Abstract:**

Early warnings of the risks of pest and disease outbreaks are becoming more urgent, with substantial increases in threats to agriculture from invasive pests. With geospatial data improvements in quality and timeliness, models and analytical systems can be used to estimate potential areas at high risk of yield impacts. The development of decision support systems requires an understanding of what information is needed, when it is needed, and at what resolution and accuracy. Here, we report on a professional review conducted with 53 professional agronomists, retailers, distributors, and growers in East Africa working with the Syngenta Foundation for Sustainable Agriculture. The results showed that respondents reported fall armyworm, stemborers and aphids as being among the most common pests, and that crop diversification was a key strategy to reduce their impact. Chemical and cultural controls were the most common strategies for fall armyworm (FAW) control, and biological control was the least known and least used method. Of the cultural control methods, monitoring and scouting, early planting, and crop rotation with non-host crops were most used. Although pests reduced production, only 55% of respondents were familiar with early warning tools, showing the need for predictive systems that can improve farmer response.

## 1. Introduction

The recent incursion of fall armyworm (FAW) (*Spodoptera frugiperda*) into maize producing communities across sub-Saharan Africa has undermined efforts to increase agricultural productivity [1]. The impact of invasive, long-flying pests such as FAW and the desert locust (*Schistocerca gregaria*) has far reaching implications on the sector, particularly because they disrupt ecosystem processes, threaten biodiversity, and burden agricultural systems with control efforts on which large portions of communities rely [2]. FAW causes widespread and intense yield losses to maize, sorghum, forage grasses, turfgrass, rice, cotton, and peanuts in its native regions [3,4]. Agricultural investments in improved seeds, fertilizer, and other agricultural inputs meant to increase yields are threatened due to the incursion of these invasive species, affecting already vulnerable producers’ food security and livelihoods [5].

FAW is a particularly difficult invasive threat to crop production in Africa because of its ability to feed on over 80 different crops. Although it has a preference for maize, it can also consume sorghum, rice, sugarcane, cabbage, groundnut, soybean, cotton, pasture grasses, and others [6]. As moths, FAW can spread locally between fields or across large areas quickly, using winds aloft to move from one area to another. FAW can also persist throughout the year whenever host plants are available. Particularly in regions with bimodal rainfall patterns such as in Kenya, FAW can persist and increase in prevalence throughout the year [7].

Research on the drivers of FAW prevalence has gained momentum since its spread into food-insecure agricultural areas of Africa and Asia [8]. Drivers of FAW distribution and prevalence used in models include land use, agro-climatic variables, and the presence of annual crops [9]. The FAW moth is migratory and can take advantage of winds aloft to travel long distances from one cultivated area to another, causing abrupt and unexpected infestations. Farmer responses to FAW invasion are varied, and range from changing the crops grown or their rotation, to the use of pesticides, early planting, applying sand and ash to young plants, frequent weeding, and hand-picking of larvae [10]. 

Modeling the impact of agricultural insect pests on production continues to be a challenge, both for the development community as well as for the modeling community due to the complexity of the agricultural system. Processes involved in pest outbreaks include variations in crop growth, crop susceptibility, weather, and insect pressure, which interact and evolve on different, non-linear trajectories [11]. Understanding what information is needed and can be used by institutions and organizations working in affected regions can help researchers focus their work and develop appropriate systems and processes. 

Information on the prevalence and intensity of fall armyworm impacts on food production can be estimated from models that use geospatial parameters, such as land use, environmental conditions including rainfall intensity and amount, and cultural practices such as biomass burning. Koffi et al. (2020) surveyed maize farms across Togo and Ghana to determine FAW larval prevalence during the season over three years. A conclusion of this study is that larval populations and infestation levels were falling during the study period due to intervention efforts as well as adverse environmental conditions. Garcia et al. (2019) used entomological models that incorporate parameters of energy absorption and food availability to predict egg stage, larval stage, and pupal stage population densities. These approaches show how monitoring and modeling can be used together to improve the information for farmers and other value chain actors on the timing of assistance and recommendations for appropriate management strategies [7]. 

Predictive models that use environmental conditions and observations have been used to provide critical information to improve decision making through an early warning system (EWS) [12]. Allen-Sader et al. (2019) describe a sophisticated EWS that is in place that warns of wheat leaf rust diseases in Ethiopia. Using a mobile-phone-enabled open data kit (ODK) field survey, 1000 standardized field surveys were undertaken by expert pathologists to identify wheat rust prevalence and to create training data for a predictive spore dispersion model. Through a multi-partner collaboration, this model was used to predict wheat rust outbreaks and operationalize an EWS for the region. This project provides policymakers, extension agents, and farmers with timely, actionable information on wheat rust prevalence and potential actions that could be taken in response to the disease. The research found that an ongoing problem is that farmers do not consistently apply fungicidal controls in response to wheat rust advisories provided in the EWS, despite the product being available in the region and the information being timely and actionable [13]. Deciding when to warn a farmer of an impending epidemic, who in the agriculture value chain should be warned [14], and what other information should be provided, such as where the farmer could find ‘plant doctors’ in the region [7], is a key question when designing these systems. 

### 1.1. Existing Early Warning Systems for Invasive Pests in Africa 

There are two primary early warning systems for invasive flying pests in eastern Africa—the eLocust system [15] and the FAW Monitoring and Early Warning System (FAMEWS) [16]—both of which are funded by the United Nations Food and Agriculture Organization (FAO). These systems rely upon a mobile application or dedicated satellite-connected tablet that allows for the collection, processing, storing, and disseminating of observational data on pest presence and impact across wide areas. These measurements are made by farmers, agronomists, technicians, or others working in agriculture, such as national survey and control officers. 

Constraints that both the FAMEWS and eLocust systems suffer is the long lag-time between when the data are collected and analyzed and when warnings of potential impacts are delivered to farmers, retailers, and input providers in the region. Although the promise of these systems is substantial, the raw observational data need to be used in a way that maximizes their utility for decision makers. 

### 1.2. Background on the Syngenta Foundation

This research was funded by the Syngenta Foundation for Sustainable Agriculture (SFSA) to ascertain participants in the agriculture value chain’s interest in information about FAW prevalence and impact. The SFSA has been working for the past 20 years to support smallholder farming and food systems through market development and delivery of agricultural innovations while building capacity across the public and private sectors [17].

The Foundation implements interventions across agricultural services, agricultural insurance solutions, and provides access to high-yielding seeds. It does this through investment in private and non-profit organizations via an innovation pipeline, which includes research, development investing, scaling up, and taking products to market. Each of its partners has different implementation models and reporting needs, depending on their characteristics. SFSA has large volumes of data gathered from a variety of its partners, some of which are commercially oriented, and some of which are governmental- or policy-focused. Due to the diversity of contexts in which SFSA works, the organization is highly decentralized; therefore, each of its country-level teams works independently and focuses on developing the best possible solutions for local realities. 

Here, we worked with the Kenya SFSA team to identify key actors working on smallholder agriculture systems with which they have direct contact, who work on supporting smallholder maize farmers via their programs. The SFSA has a direct relationship with each of the respondents’ organizations; therefore, the respondents were not randomly chosen, but were selected according to their engagement with the appropriate agriculture sector. The Foundation has a system that requires partner organizations to report on the outputs of the operations and the outcomes of the activities of each funded activity [17]. For this study, we selected respondents in partner organizations that have a clear connection with the community and are strategically positioned to implement a potential forecasting system on FAW. Messages delivered via an EWS should be designed so that the content and format will result in actionable, usable information for the agricultural community.

### 1.3. Purpose and Objectives 

The research presented here is focused on determining the optimal timing, content, and recipient of information on outbreaks of new maize pests such as FAW. Our hypothesis is that information on future risks for large-scale outbreaks can be very useful for planning appropriate responses across a variety of actors in the agriculture value chain. As a first step before investing in setting up a new operational EWS for invasive pests in eastern Africa, we conducted a review of professionals engaged with the Foundation to determine their needs.

We present a professional review of experts involved in development programs funded by the SFSA, whose investments in seed development, responses to pest threats, and risk reduction are conducted through private and public sector actors in East Africa. Even with effective predictive models, connecting model output to farmers, input providers, and retailers that can support decision making on how to prevent outbreaks and what to do when they occur remains an issue. Migratory, transboundary pests such as FAW and the desert locust are very challenging to local growers, because they have little insight as to when or where they may be affected, and to what degree. The results of this study have implications for agroecological systems that may be affected by many other invasive pests and diseases.

We conducted a comprehensive survey of the use of production techniques, strategies, and useful approaches to responding to pest threats within a group of agronomists, managers, and others involved in farming. We use this information to design a system that can provide advice and recommendations to actors within an agriculture development program via soliciting the needed information via a quality assurance assessment review. Geospatial tools and mobile applications can be transformative to warn of potential problems and communicate actions, but only if the information provided is relevant, salient, reliable, and timely. We first describe the development program through which the review was conducted, and describe the data collected and participants involved; then, we present the results from agronomists/growers and from those in the non-farmer segment. We then discuss these results and their implications for agricultural development programs. 

## 2. Data and Methods

Through the SFSA professional network, we identified agronomists, distributors, managers, and others working in agriculture. Each was sent a letter along with a Word document with over 50 questions (the full questionnaire is available in the Supplementary Materials). The focus of the questions was how to best understand the Foundation’s responses to the informational needs of participants in its programs on invasive pests. Below, we describe the characteristics of the respondents and more details on the survey instrument. 

### 2.1. Professional Survey Respondents

A professional review of 53 agricultural professionals in Kenya (81%), Ethiopia (2%), Rwanda (7.5%), Tanzania (7.5%), and Uganda (2%), some of whom work with the Syngenta Foundation for Sustainable Agriculture (SFSA), was carried out. The response rate was 63% (53 returns from 85 questionnaires that were distributed). A total of 23 of the 53 respondents worked for an association and represented groups of farmers, millers, retailers, seed companies, agronomists, cereal farmers, service providers such as for plowing or harvesting, pest control products, and others. The associations varied in size, from approximately 60 (United Millers and Farmers Association) to 2.1 million (Kenya National Farmers’ Federation) members, and participated in the agriculture value chain in East Africa [18].

**Figure 1:**
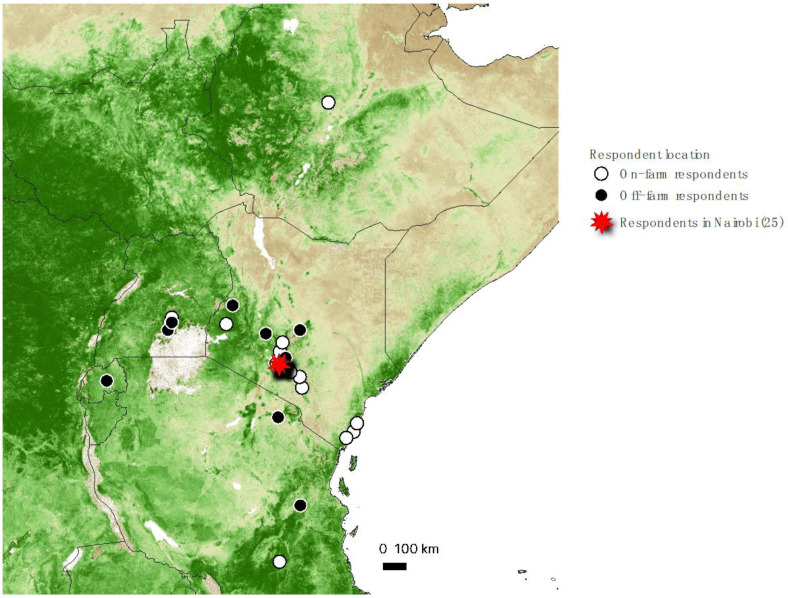
Location of respondents in East Africa, with 25 located in Nairobi, Kenya.

Respondents of the survey were 75% male, and were highly educated: 45% had a doctorate, 28% a masters’, and 9% a bachelors’ degree, with the remaining having professional diplomas and secondary degrees. Approximately half the respondents worked as agronomists, farm managers, or were involved in research farms that created new hybrid maize varieties or the efficacy of new agronomic practices in Kenya, and the other half were involved in distribution, retail, government, or program development in various capacities. Figure 1 shows the location of work for the respondents.

The respondents of the survey stated their professions to be entomologists, research scientists, plant breeders, managers of associations and research institutes, coordinators of policy and government affairs, and managers of research on pests, integrated pest management and maize breeding. The respondents were primarily senior personnel and decision makers.

### 2.2. Methods of Data Collection

The questionnaire involved asking specific questions regarding how information on pest outbreaks could be used within the respondents’ activities. The questions focused on determining the scope and timing of a potential geospatially informed information data product on pest outbreak probability from models. The survey was distributed via email and was filled out using a Word document. Table 1 shows the types of questions included in the survey, the answer type in the document, and the purpose of the question area. All respondents were able to respond to all questions, although only half of the respondents answered questions regarding on-farm activities or pests. Some of these responses were on institution- or corporate-owned farms, and some farms were owned by the respondent personally. We transformed the digital Word document questionnaire into an Excel spreadsheet for analysis. Each of the questions was analyzed and prioritized in the results.

## 3. Results

Results show that respondents have a high interest in early warning systems that can provide early warning of pest outbreaks that affect agricultural production. There was significant interest in early, actionable information that includes interactive pest distribution maps as well as management recommendations. Pests were a significant source of concern, but also one of commercial interest. We first describe the farmer-level responses and the management strategies currently employed; then, we describe the recommended design of the system.

### 3.1. Impact of Pests on Productivity in the Agriculture Sector

**Figure 2:**
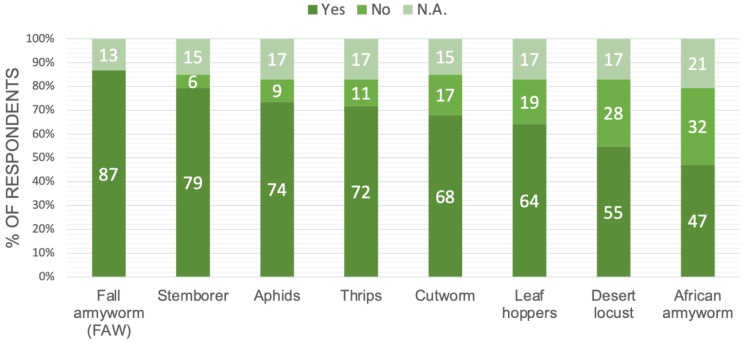
Percentages of respondents reporting pests in cereal crops in eastern Africa.

We found that multiple pests threaten cereal production in East Africa, with the most commonly reported pests being FAW, stemborer, and aphids (Figure 2). Of the crops listed, maize, potato, cotton, rice, cowpea, and vegetables were most commonly cultivated, although there was a large diversity of crops grown. 

Respondents stated that they saw business or programming opportunities from preventing yield loss due to pests to include:

- Development of pest-tolerant crops or breeding native genetic resistance to pests;
- Conducting efficacy trials for management control practices or products;
- Selling technologies to reduce damage including pesticides or IPM approaches of pheromones or insect traps;
- Managing yield loss due to pests is critical for sustaining and achieving production goals of the program;
- Training farmers on how to implement integrated pest management (IPM) approaches [2]; and
- Identifying potent natural enemies of invasive pests such as FAW, and training on diversified cropping systems such as push–pull [19].

**Figure 3:**
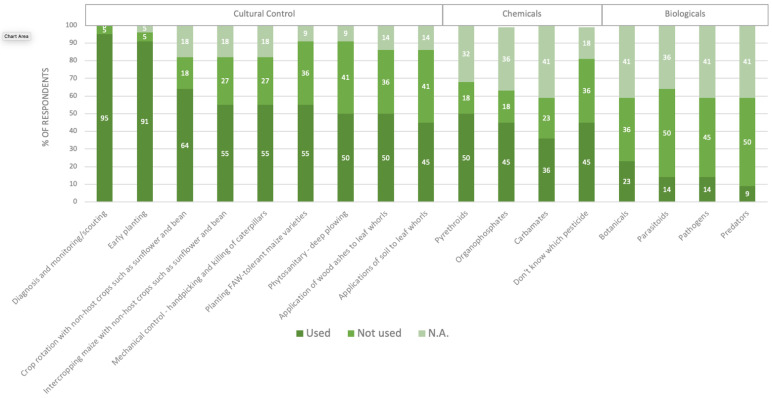
Percentages of respondents reporting different methods for FAW control in cereal crops. Respondents who did not answer the question were reported as N.A.

Of the strategies used to control FAW, 100% of the respondents stated that they used chemical control such as pesticides to control an FAW outbreak, 97% stated that they used cultural controls, 90% used IPM strategies, but only 22% stated that they used biological controls. Cultural control elements include diagnosis and scouting, early planting, crop rotation, intercropping, mechanical control or hand-picking, applications of wood ash to whorls, and the planting of FAW-tolerant maize varieties. Biological techniques are the least known and used method of FAW control, and were not widely available in our study region (Figure 3).

### 3.2. Design of an Early Warning System for FAW and other Pests

Each respondent was asked about the timing of decision making regarding the pest control measures listed in Figure 2. We found that the time needed for an outbreak warning is dependent on the control practice. Some practices, such as crop rotation, the planting of pest-tolerant varieties, implementing IPM, or conducting intercropping, require one month or more advance notice, preferably before the start of the season. Other interventions, such as applying chemical or biological controls, only require only one week or more advance notice. 

When asked if the respondent was familiar with an existing insect pest outbreak prediction tool, 55% responded yes, 34% responded no, and the remainder did not answer the question. When prompted to disclose with which tool they were familiar, the following responses were provided:

- Fall Army Worm Monitoring and Early Warning System (FAMEWS);
- The desert locust prediction and warning tools from the FAO;
- The community-based fall armyworm monitoring and early warning system, which has been piloted in five East African countries, funded by the United States via the FAO;
- Pest Risk Information Service (PRISE), supported by CABI, piloted in Ghana, Kenya, and Zambia;
- The use of species-specific pheromone traps and light lures to warn of a specific moth presence;
- Static pest distribution maps for the probability of pest presence from various sources.

All these projects work by combining pest surveillance monitoring with some simulation or predictive modeling for pest outbreaks. However, they require the ongoing support and monitoring of species-specific pheromone traps. Connecting the information gathered across very large areas to a centralized modeling and communication system requires ongoing funding and support.

All 53 respondents were asked in a freeform question to state the benefits of an FAW outbreak prediction tool to their institution or agricultural business. Overall, respondents wanted an ‘accurate’ tool that will help farmers avoid losses due to pests, and that the tool is ‘easy to use, affordable, and accessible cheaply’. Table 2 sets out all the benefits stated by the respondents and the number of times the idea was suggested. 

**Figure 4:**
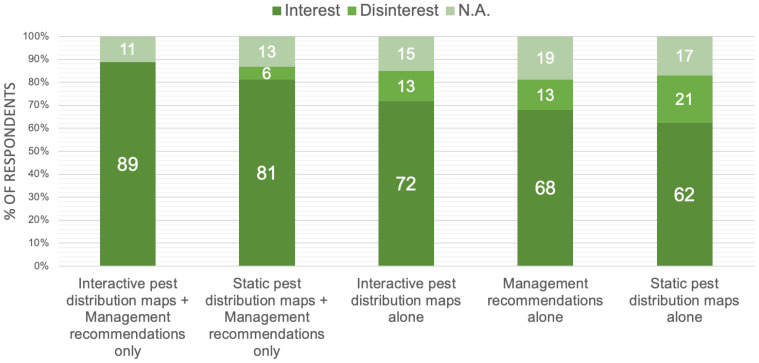
Percentages of respondents reporting which type of FAW prediction product they would be interested in when presented with the choices of static distribution maps, interactive distribution maps or management recommendations, or a combination of these.

When asked whether management recommendations were most important, or if maps would also be useful, respondents would be interested in all proposed insect pest outbreak prediction products, but would particularly like an interactive map together with FAW management recommendations (Figure 4). When asked, respondents stated that recommendations might include information on the best control methods, including what products are available beyond simply chemical pesticides and how to use them. Awareness of the latest control measures could be promoted through the prediction product and the problems of chemical buildup or biological resistance if a single product or approach is overused. Determining which IPM approach should be taken and when in the upcoming season would also be very helpful. 

Most respondents stated that the most essential information which should be provided in a prediction system was the timing of the outbreak during the season and the likely damage that it would cause, but only if these could be provided accurately. Knowing when an outbreak may peak, its intensity, and its location is essential to determining the geographic coverage of where interventions should be planned. Predicting these could greatly enhance reducing their impact on overall agricultural productivity and reducing the cost of inappropriate or unnecessary interventions. 

## 4. Discussion

This paper provides evidence from a structured review of 53 agronomists, engineers, development experts, farmers, managers, and agriculture professionals in East Africa who are working in the agricultural development field. We found extensive interest in an operational early warning system for pests that would be able to provide an early warning of pest outbreaks. Growers, scientists, agriculture companies, and non-profits can access information about pest prevalence from national plant protection organizations across the region, but these organizations rarely provide early warnings that are spatially explicit [2]. The information is rarely sufficiently timely to provide appropriate guidance on management and decision making, however. 

Previous work on early warning systems has noted the need for pest early warning systems to be continuously updated and monitored. For example, the eLocust system set up by the FAO in 2016 relies upon individual pest reports via mobile devices, along with monitoring and responses by multiple agencies across very large areas [15]. Quansah et al. (2010) pointed out the difficulties and costs of setting up automated systems that can continuously monitor pest threats. Both Quansah and Cressman recognized the limitations of the real-time data collection and transmission of pest prevalence and impact, given the need for reporting by large numbers of people across multiple institutions and regions [20]. By using modeling and prediction of the probable impact, the reliance on real-time collection can be minimized, because most predictive models rely upon historical pest prevalence information, seasonal climate information, and current weather conditions to provide forecasts [21]. Rapidly changing climate drivers and the introduction of new maize varieties mean that these models need to be based on information gathered and incorporated into predictive models within one year of use. Leveraging mobile phones, ODK survey systems, and the delivery of EWS messaging via chat platforms are all critical strategies for information relevance, timeliness, and salience [22]. 

Results show that among the agriculture professionals surveyed, chemical and cultural controls were the most common strategies for pest control, and biological control was the least known and least used method. This reflects the widespread availability of pesticides in East Africa, despite the lack of specific compounds for new invasive pests such as the fall armyworm. Sharanabasappa et al. (2020) found that despite the low efficacy of a variety of chemical pesticides against FAW in maize field trials, yield was doubled over the control due to the suppression of populations in the early part of the season [23]. These rather poor results are on par with cultural controls for this pest [24]; thus, more research is needed to identify new ways of controlling the development and infestation of these invasive pests.

We show with this research that there is substantial demand for spatially and temporally specific warnings, as well as for specific recommendations as to the actions that should be taken to respond. Our respondents reported considerable interest in increased intelligence on potential pest threats in the coming weeks and months. Information on where and when hotspots of pest pressure would emerge has considerable value, even for respondents working in the development space with smallholder farmers. Responses to pest threats, which could include changing crop type, planting date, input investments, or the use of cultural practices to reduce yield impacts, should be included in any system that provides pest forecasts [25]. Accelerating the knowledge and use of affordable and effective interventions to current and emerging pest threats across middle-income countries such as Kenya can be facilitated with an early warning system that focuses on the next few weeks instead of current conditions.

## 5. Conclusions

The results showed that respondents were interested in FAW outbreak predictions with high spatial granularity—both at local and county levels. This would help decision makers determine how to reduce risks from pests and expenses from pest management, and to use more effective approaches to manage pests. Predicting outbreaks before they occur will help farmers invest when necessary, but not over-apply pesticide when the risk is low. Early detection, rapid assessment, and rapid response to potentially crop-destroying pests and diseases are essential to effectively guard against yield loss [26]. Therefore, spatial and temporal specificity of the EWS information is essential for improved response.

Eastern Africa has multiple rainy seasons and farmers plant and harvest cereals throughout the year; therefore, maize is particularly susceptible to FAW outbreaks. Without controls, farmers could lose a substantial portion of their main staple food, causing food security problems [27]. Therefore, warnings of high pest pressure in the coming season would be valuable for decision makers across the value chain—input providers, retailers, and farmers need warning to respond to different conditions. Off-season prediction of the risk of harmful fall armyworm and other invasive pests in a specific region in the upcoming season can be two weeks to one month before the season starts. An EWS should be robust, with simple messages, and ideally, combine warnings and advice on mitigation options. Table 3 summarizes our results which indicate that at different stages of the value chain, users need different prediction horizons and time periods. Although planning for integrated pest management, the use of different varieties, switching to new crops, and other farming interventions require clear indications that the fall armyworm or other pests will be a problem before the start of the season, many decisions could be made to respond to an emerging outbreak during the season.

The respondents appreciated the benefits of a pest and disease outbreak warning system, with a number of the respondents describing the need for additional training, research into the interactions between invasive pests and natural predators in each agroecosystem, and helping smallholders gain access to modern farming technologies that enable farmers to overcome pests damage. Participants also noted that early warning could help farmers reduce the cost of production through anticipation of the severity of the problem.

Respondents would like the tool to be simple, easy to access and use, timely, and accessible through mobile phones. In collaboration with research and development partners such as CABI, the Food and Agriculture Organization, the Kenya Agricultural and Livestock Research Organization (KALRO), and the International Centre of Insect Physiology and Ecology (ICIPE), an early warning system can be integrated into training curriculums that include pest identification, diagnostic tools, pest biology/damage symptoms and ecology, and monitoring.

Our results show that much more work needs to be carried out to improve knowledge of the benefits of integrated pest management [24], the use of biopesticides and parasitoids [28], and management strategies such as early planting and proper fertilization that reduce the susceptibility of an agricultural system [29]. Invasive pests and diseases that can move into an area from over long distances are becoming increasingly common and economically important [30]. These approaches need to be implemented across both smallholder and commercial farming systems to be effective. An early warning system can be instrumental in communicating the risk to farmers while ensuring that response options are available when needed.

## Figures and Tables

**Table 1:** Characteristics of questions in a survey on information on prediction tool for FAW outbreak in East Africa. Complete questionnaire is available in the Supplementary Materials.

Question Topic	Answer Format Details	Purpose of Question Topic
Personal information	Name, address, place of work	Determine the location of work, type of expertise, age, and education characteristics
Institutional information	Name of organization, type of position, current role	Understand respondent expertise and ability to understand and plan for FAW system use
Pest business opportunities	Freeform request to speculate on potential business opportunities	Determine how an FAW Early Warning System could be used within the organization, what the organization does with information on pests
On-Farm Respondents		
Size of area cultivated	Area in cultivation, either privately or as part of the institution	Diversity of farming system that survey addresses
Pests experienced in work	Ranking of pests, damage experienced from pests, crop growth stage most affected, percent of resources	Understanding FAW importance when compared with other pests
Type of pest management used	Ranking of strategies used, type of responses	Understanding when or if cultural, chemical, biological or integrated pest management approaches were used to control pests
Detailed questions about management approach	Effectiveness and use of various management approaches	Understanding of pest management within each organization and during which crop growth stage
Timing of decision making	Timing of decision making on each pest management approach	Understanding of how far in advance each organization needs before deciding on a pest management approach
On and Off-Farm Respondents		
Familiarity with other FAW prediction tools	Asks to list tools or approaches familiar with	Analysis of demand for additional methods on FAW and other pest management approaches
Characteristics of a pest prediction tool	Asks respondent to select potential product elements such as static maps, dynamic maps, and recommendations	Helps to determine what FAW information would be most useful for institutions represented
How FAW prediction could help in core business	Freeform text entry of benefits of an FAW prediction tool	How providing FAW prediction tool can help with accelerating business performance across industries and applications

**Table 2:** Responses to freeform question to respondents regarding the benefits of an FAW outbreak prediction tool to their business or institution.

Responses	No. of Respondents	Percentage Score (%)
Facilitate planning of FAW control measures	40	20
Opportunity to obtain knowledge and training in effective FAW management	21	10.5
Facilitate timely procurement of effective FAW control products	21	10.5
Facilitate selection of the crop and variety for reduced impact from FAW attack	14	7
Facilitate informed decision-making on FAW policy, practice, and research	15	7.5
Facilitate estimation and prediction of expected harvest considering an FAW outbreak	12	6
Empower advisory service providers with information on FAW	12	6
Inform the type of management tool to be applied against the FAW (whether mass trapping, pesticide sprays, or biological control)	10	5
Facilitate budgeting for FAW control measures (e.g., pesticide purchase)	9	4.5
Help delineation of affected areas and focusing management efforts of FAW	8	4
Enable carrying out timely scouting for FAW damage	7	3.5
Facilitate decisions on the time of planting the selected crop.	6	3
Use of data to develop pest models for pest prediction	6	3
Facilitate neighboring farmers to effect community level FAW control	3	1.5
Facilitate prediction of markets for grain and agricultural inputs	3	1.5
Facilitates the development of an effective crop rotation plan	3	1.5
Facilitate making of well-targeted, pre-emptive sales and distribution of FAW control by manufacturers and agro-dealers	5	2.5
Facilitates choice of which IPM method to use	2	1
Facilitate prediction of where to source timely grain imports from the region	2	1
Allow for the development of a county- or district-level pest risk map	1	0.5
Total Responses	200	100

**Table 3:** Results on different management options derived from survey results.

	Input Distribution		On-Farm Management	
	Prediction Time	Spatial Resolution	Prediction Time	Spatial Resolution
Cultural control	3–6 months	Low	3–6 months	Low
Biological control	1–2 months	Medium	1 month	High
Chemical control	1 month	Medium	1–2 weeks	High

## Data Availability

Data from this study are available upon request.

## Supplementary Materials

The following supporting information can be downloaded at: https://www.mdpi.com/article/10.3390/insects13030232/s1, Text S1. Questionnaire used in professional review.
