# Supplementary material for: Designing a Pest and Disease Outbreak Warning System for Farmers, Agronomists and Agricultural Input Distributors in East Africa
Source: Insects. 2022 Feb 26;13(3):232. doi: 10.3390/insects13030232 (PMC8948835; doi:10.3390/insects13030232)
Supplement: Supplementary file 1 [file insects-13-00232-s001.zip › insects-1520188-supplementary.pdf]

Questionnaire for Assessing Stakeholder Control and Needs from Prediction Tools for FAW Outbreaks in Eastern Africa

Questionnaire for Researchers and Farmers

Q1. Questionnaire No …………………………..

Q2. Date: ………

Location: ………..

Q3. Village / Town………

Q4. District……..

Q5. County/Province……………………………………………………………………………………………………

Q6. Country………

I. Respondent

Q7. Name: ……

Q8. Gender: 1. M…………..2 F………...

Q9. Age…………………………………………… (Optional) ………………………………………………………………..

Q10. Education level:

Primary……… Secondary……… Bachelors………. Masters ……….. PhD……… (Optional) ……………..

Q11. Position………

Q12. Role in your institution……………………………………………………………………………………..

Contacts:

Q13. Physical address……………………………………………………………………………………………………..

Q14. Telephone Numbers……………………………………………………………………………………………

Q15. Email Address………

II. Institution

Q16. Name ………

III. Core agricultural business

Q17. Is your institution an Apex body or Head of an Association? …Yes ………..No…………………

If yes it's an Apex body:

Q18. Who are the members ………………………………………………………………………………………

Q19. How many members does your Apex Body have? …………………………………………………………

Q20. What do the members do? ………………………………………………………………………………………

If No i.e. it's a standalone institution:

Q21. What is its Core business……………………………………………………………………………………………
………..

Q22. What is its location (if different from respondent location)…………………………………………
………..

What is its contacts (if different from respondent contacts)?
Q23. Physical address…………………………………………………………………………………………………….
……
Q24. Telephone Numbers…………………………………………………………………………………………………
Q25. Email Address…….

IV. Core Business

Q26. How much crop land area do you plant during the main season on average? ………….Ha

What crop commodities do you or your member grow / deal with?

**Commodity**	Q27. **Yes/No**	Q28. **Area Planted during main season 2020**	Q29. **Did you observe any FAW damage during the season 2020? Yes/No**
Maize			
Millet			
Wheat			
Potato			
Soybean			
Cowpea			
Peanuts			
Sorghum			
Rice			
Sugarcane			
Vegetables			
Cotton			

Q30. What business opportunities do you have from insect pests?

1. ………
2. ………
3. ………
4. ………

V. Insect Pests

Do you see any of the following insect pests on your farm?

**Insert pest**	Q31. **Presence (Yes/No)**	Q32. **Stage of growth when pest appears**	Q33. **Rank in terms of damage (1=highest, 5= lowest)**	Q34. **Estimate loss (%) of expected harvest)**
Cutworms				
Stem borers				
Desert locusts				
African armyworm				
Fall armyworm				
Thrips				
Aphids				
Leaf hoppers				
Others (specify)				
Others (specify)				

Q35. How much average crop loss do you suffer from FAW damage per year? …………………%

VI. FAW Control Measures

What strategies do you current use to control FAW outbreak?

**FAW Control strategies**	Q36. **Do you use each strategy? Yes / No**	Q37. **Effectiveness of control (1= Most effective, 5= Least Effective)**	Q38. **What stage of crop growth do you apply this strategy? (pre-planting, seedling, pre-flowering, post-Flowering)**
Cultural control			
Chemical control			
Biological control			
Integrated Pest management			
Other (specify) ………………………			

If Yes for cultural control, which cultural control methods do you current use to control FAW outbreak?

**FAW Control strategies**	Q39. **Do you use each method? Yes / No**	Q40. **Effectiveness of control (1= Most effective, 5= Least Effective)**	Q41. **What stage of crop growth do you apply this strategy? (Pre-planting, seedling, pre-flowering, post-Flowering)**
**Diagnosis and monitoring/scouting**			
**Phytosanitary – deep plowing**			
**Early planting**			
**Crop rotation** with non-host crops like sunflower and bean			
**Intercropping** maize with non-host crops like sunflower and bean			
**Mechanical control - handpicking** and killing of caterpillars			
Application of **wood ashes** to leaf whorls			
Application of **soils** to leaf whorls			
Planting FAW **Tolerant maize varieties**			
Others (specify) …………………………………			

If Yes for chemical control, which chemical control methods do you current use to control FAW outbreak?

**FAW Control strategies**	Q42. **Do you use each method? Yes / No**	Q43. **Effectiveness of control (1= Most effective, 5= Least Effective)**	Q44. **What stage of crop growth do you apply this strategy? (Pre-planting, seedling, pre-flowering, post-Flowering)**
Pyrethroids			
Carbamates			
Organophosphates			
Don't know the pesticide category			
Others (specify) ……………………………			

If Yes for biological control, which biological control methods do you current use to control FAW outbreak?

**FAW Control strategies**	Q45. **Do you use this method? Yes / No**	Q46. **What product(s) do you use?**	Q47. **Effectiveness of control (1= Most effective, 5= Least Effective)**	Q48. **What stage of crop growth do you apply this strategy? (pre-planting, seedling, pre-flowering, post-Flowering)**
Parasitoids				
**Predators**				
**Pathogens**				
**Botanicals**				
Others (specify) ………………………… …………………				

Q49. If yes for Integrated Pest management, please describe the combination of strategies and methods that you currently use to control FAW?

………
………
………
………

Q50. What is the lag time i.e. how far in advance of potential outbreaks do you need to make decisions to change a FAW management practices?

**FAW Control strategies**	**When do you decide to use this method?**				
	1-3 Days	1-week	2-weeks	1-Month	>1-month
Crop rotation					
Chemical pest control					
Intercropping					
Biological control					
Seed treatment					
Tolerant varieties					
IPM measures					
Other (specify) …………………………………					
Other (specify) …………………………………					

VII. FAW outbreak prediction tools

Q51. Are you familiar with any insect pest outbreak prediction tools?
Yes……………….No……………..

Q52. If Yes, Which one(s)?
……………………………………………………………………………………………………….

What insect pest outbreak prediction products would you be interested in?

**Product**	**Q 53. Yes / No**	**Q 54. Rank (1= Highest priority)**
Static pest distribution maps alone		
Interactive pest distribution maps alone		
Management recommendations alone		
Static pest distribution maps + Management recommendations only		
Static pest distribution maps + Management recommendations only		
Others (Specify) ………………………………………………………………..		

Q55. If FAW outbreak prediction tool was available, what are the three important message(s) would you like to receive on the outbreak?

1. ……
2. ……
3. …….

If FAW outbreak prediction tool was available, what three geographic areas would you like it to cover?

**Geographic area**	Q56. **Rank (1= highest priority, 5 = lowest priority)**	Q57. **Please state the reason for your ranking for each area**
My own farm		
Administrative sub-location / location		
Administrative sub County (Former division)		
Administrative County (District)		
Administrative Region (Former Province)		
Country level		

If FAW outbreak prediction tool was available, how early would you like to receive the message for you to make adjustments before the impending outbreak both during off-season and in-season? Rank your preferences in the table below:

	**Off-season prediction**	Q58. **Rank your preference (1=Highest)**	**In-season prediction**	Q59. **Rank your preference (1=Highest)**
1	Warnings for today		Warnings for today	
2	One day in advance		One day in advance	
3	2-3 days in advance		2-3 days in advance	
4	One week in advance		One week in advance	
5	Two weeks in advance		Two weeks in advance	
6	One month in advance		One month in advance	
7	2-3 Months in advance			
8	More than 3 months in advance			

Q60. List the 5 most important ways in which FAW outbreak prediction messages would benefit you in your core business.

1 ……

2 ……

3………

4 ……

5 ……

Q61. What else would you like to advice those who may wish to develop FAW outbreak prediction tools?

1. ……
2. ……
3. ………..
4. ………..

Thank you very much for participating in this survey
